# The Plant Pathology Challenge 2020 data set to classify foliar disease of apples

**DOI:** 10.1002/aps3.11390

**Published:** 2020-09-28

**Authors:** Ranjita Thapa, Kai Zhang, Noah Snavely, Serge Belongie, Awais Khan

**Affiliations:** ^1^ Plant Pathology and Plant‐Microbe Biology Section Cornell University Geneva New York 14456 USA; ^2^ Cornell Tech 2 W Loop Road New York 10044 USA

**Keywords:** apple orchards, computer vision, convolutional neural network, disease classification, machine learning

## Abstract

**Premise:**

Apple orchards in the United States are under constant threat from a large number of pathogens and insects. Appropriate and timely deployment of disease management depends on early disease detection. Incorrect and delayed diagnosis can result in either excessive or inadequate use of chemicals, with increased production costs and increased environmental and health impacts.

**Methods and Results:**

We have manually captured 3651 high‐quality, real‐life symptom images of multiple apple foliar diseases, with variable illumination, angles, surfaces, and noise. A subset of images, expert‐annotated to create a pilot data set for apple scab, cedar apple rust, and healthy leaves, was made available to the Kaggle community for the Plant Pathology Challenge as part of the Fine‐Grained Visual Categorization (FGVC) workshop at the 2020 Computer Vision and Pattern Recognition conference (CVPR 2020). Participants were asked to use the image data set to train a machine learning model to classify disease categories and develop an algorithm for disease severity quantification. The top three area under the ROC curve (AUC) values submitted to the private leaderboard were 0.98445, 0.98182, and 0.98089. We also trained an off‐the‐shelf convolutional neural network on this data for disease classification and achieved 97% accuracy on a held‐out test set.

**Discussion:**

This data set will contribute toward development and deployment of machine learning–based automated plant disease classification algorithms to ultimately realize fast and accurate disease detection. We will continue to add images to the pilot data set for a larger, more comprehensive expert‐annotated data set for future Kaggle competitions and to explore more advanced methods for disease classification and quantification.

The U.S. apple industry, annually worth $15 billion, experiences millions of dollars in annual losses due to various biotic and abiotic stresses, ongoing stress management, and multi‐year impacts from the loss of fruit‐bearing trees. Over the growing season, apple orchards are under constant threat from a large number of insects, as well as fungal, bacterial, and viral pathogens, particularly in the northeastern United States (Fig. 1). Depending on the incidence and severity of infection by diseases and insects, impacts range from unappealing cosmetic appearance, low marketability, and poor quality of fruit, to decreased yield or complete loss of fruit or trees, causing huge economic losses (Sutton et al., 2014). Early pest and disease detection are critical for appropriate and timely deployment of disease and pest management programs (Bessin et al., 1998). Disease and pest risk prediction models and management programs are developed based on incidence, severity, and timing of infection, taking into account current and forecasted weather data (Gadoury and MacHardy, 1986; MacHardy et al., 1993; Trapman and Polfliet, 1997; Stewart et al., 1998). Incorrect and/or delayed diagnosis and treatment can lead to rapid spread of diseases, and even small instances of insect damage and disease can quickly become a larger and costlier problem when pathogens multiply rapidly, particularly under favorable environmental conditions. In addition, misdiagnosis can result in either over‐ or under‐use of chemicals, leading to the emergence of resistant pathogen strains, increased production costs, increased environmental and health impacts, or potentially to a significant outbreak. Modern high‐density apple orchards, which are usually made up of a few highly susceptible cultivars, are particularly vulnerable to the rapid spread of pathogens, potentially killing the entire orchard (Peil et al., 2009).

**Figure 1:**
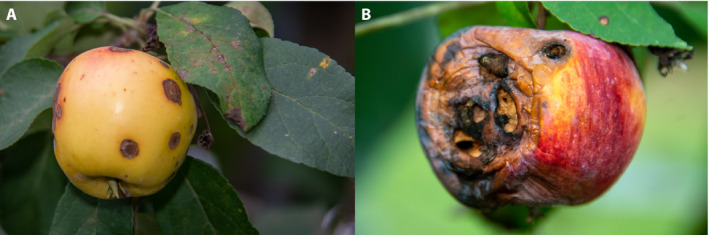
Examples of common and economically important fungal diseases in an apple orchard impacting cosmetic appearance, fruit quality, and yield: apple scab (*Venturia inaequalis*) on fruit and leaves, and cedar apple rust (*Gymnosporangium juniperi‐virginianae*) on leaves (A); black rot and frogeye leaf spot (*Sphaeropsis malorum*) on fruit and leaves (B).

Currently, disease and pest detection in commercial apple orchards relies on manual scouting by crop consultants and service providers (Judd et al., 2017; Li et al., 2017; Deutsch and Guédot, 2018). Unfortunately, very few experienced scouts are available, forcing them to cover many large orchards within a narrow time frame. Scouts require a great deal of expertise and training before they can be efficient and accurate in diagnosing an orchard. Generally, they are first trained using images of disease symptoms and insect damage, but due to the presence of a great number of variables in an actual orchard, they need considerable time to familiarize themselves with the many symptom classes caused by either the age and type of infected tissues or the stage of the disease or pest cycle, as well as by changing weather, geographical variances, and cultural differences. Experienced scouts also generally establish a pattern for random sampling to avoid visually evaluating every tree, particularly in large orchards, where areas must be strategically scouted to cover the most important ones. Scouts will look for specific susceptible cultivars (e.g., ‘McIntosh’) or specific regions of the orchard (e.g., edges). Many symptoms of diseases, pests, and abiotic stresses in an apple orchard are distinct enough to differentiate based on visual symptoms alone. However, several disease symptoms look similar enough to each other that it is difficult to accurately determine their cause (Barbedo, 2014). At the same time, visual symptoms of a single disease or particular insect can vary greatly between apple varieties, due to differences in leaf color, morphology, and physiology. Specific temperatures, humidity levels, and the physiological developmental stage of a plant also play a crucial role in disease infection and insect development (Dai et al., 2019; Wöhner and Emeriewen, 2019). The shape and form of symptoms can also vary over time as the disease progresses and leaf or fruit tissue ages. In addition to the time spent in the orchard, a scout spends a significant amount of time on each client, entering the scouting report, interpreting results, and providing recommendations for action (e.g., what kind of spray schedule, spray mixture, blanket vs. spot spray, whether pruning is needed). Overall, human scouting is usually time consuming, expensive, and in some cases, prone to errors.

In recent years, digital imaging and machine learning have shown great potential to speed up plant disease diagnosis (Mahlein, 2016). The digital imaging revolution has already created tremendous opportunities in many fields of social and professional life, and much of the world has ready access to a smartphone with an integrated digital camera that can be used to capture high‐quality images of disease symptoms. Computer vision methods are being developed to make use of digital images of symptoms for disease classification (Sladojevic et al., 2016; Amara et al., 2017). These methods combine human expertise and machine learning algorithms to find relationships and visual patterns for grouping and identification. Usually, crowdsourcing platforms are used to collect images with metadata and images are later annotated by experts for training deep neural network models. Once models have been trained, unidentified images can be automatically identified using these models. In recent years, many crowdsourced image platforms have become popular among data scientists to solve complex “big data” problems. For example, Kaggle (https://www.kaggle.com) is a popular platform that organizes global competitions to solve complex data science and machine learning–related problems, awarding prizes and ranks to the winners (Iglovikov et al., 2017; Sutton et al., 2018; Mwebaze et al., 2019). Global competitions on a large‐scale evaluation platform provide the opportunity to identify the best model and creative solutions among a large number of models submitted by data scientists from around the world (Puurula et al., 2014; Sutton et al., 2018). Such competitions provide an opportunity for multidisciplinary collaborations between computer science, biological science, and agriculture to solve both basic science and agricultural issues for food security and sustainability (Mwebaze et al., 2019).

When using computer vision as a tool for precise disease identification, all potential symptom variables must be accounted for in the digital image database (Barbedo, 2018). For example, image capture conditions for a tree must include multiple positions and angles of infected tissue, a variety of ambient light levels, various capture sensor types, and different season and weather results, in addition to illustrating the effect of each of the different diseases on fruits and leaves of various ages (Figs. 2, 3). This makes it difficult, although attractive and theoretically possible, for symptom‐based computer vision models to automatically classify disease symptoms with a high degree of accuracy without supervision by an expert plant pathologist (Kamilaris and Prenafeta‐Boldú, 2018). A large number of high‐quality real‐life images of disease and insect damage symptoms can be collected and expert‐annotated to train computer vision models with high prediction accuracy (Barbedo, 2018). Deep convolutional neural network (CNN) models and other machine learning models have been previously developed and tested to classify diseased leaf images of crop plants taken in controlled and/or uniform settings (Dubey and Jalal, 2016; Mohanty et al., 2016; Liu et al., 2018).

**Figure 2:**
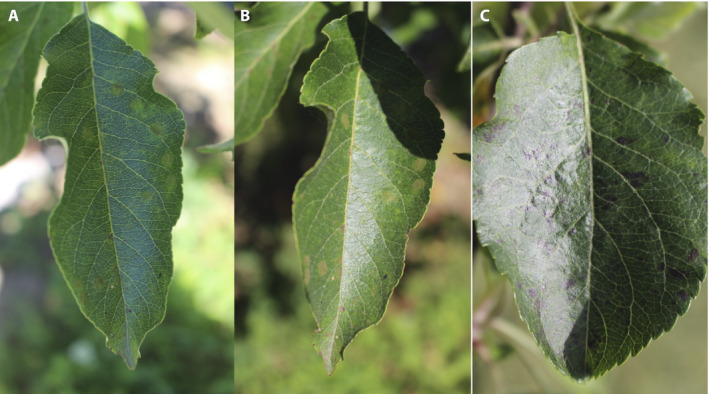
Images of disease symptoms on apple leaves captured under different light conditions: indirect sunlight on leaf (A), direct sunlight on leaf (B), and strong reflection on leaf (C).

**Figure 3:**
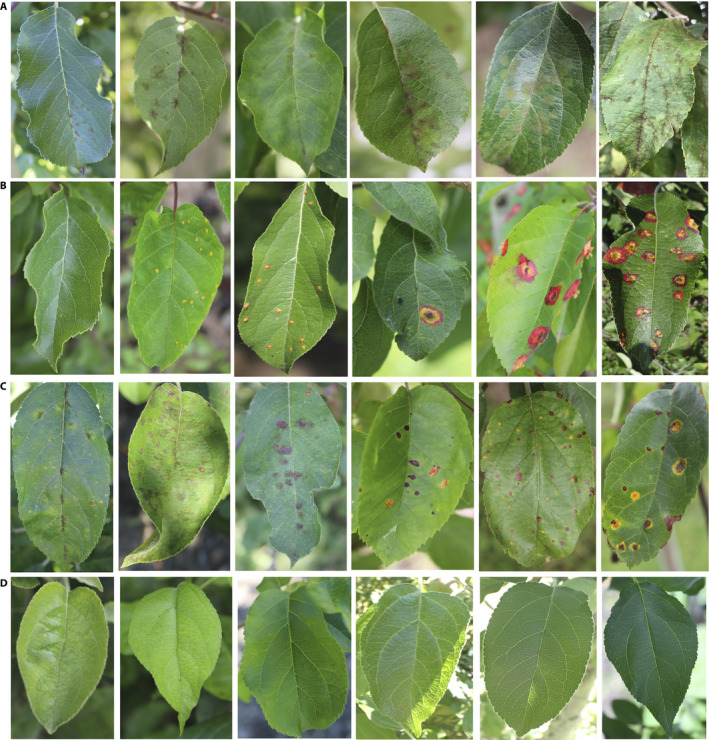
Sample images from the data set showing symptoms of cedar apple rust (A), apple scab (B), multiple diseases on a single leaf (C), and healthy leaves (D). Images of symptoms were captured using a digital camera and a smartphone in a research orchard from a large number of apple cultivars at Cornell AgriTech (Geneva, New York, USA) in 2019. Images show variation in light conditions and early to late stages of disease symptoms on young and old leaves in different disease categories.

Recent publications (Barbedo, 2014; Pethybridge and Nelson, 2015; Atoum et al., 2016) have explored the use of computer vision to identify diseases in crops at a more complex level. For example, a CNN‐based approach was used to detect and distinguish leaves infected with Cladosporium speckle disease from healthy leaves under challenging photographic conditions, including complex backgrounds and different resolutions, orientations, and illumination levels (Amara et al., 2017). Pethybridge and Nelson (2015) developed a digital image–based, portable, interactive, and semi‐automatic smartphone application to distinguish diseased tissues from healthy tissues. For assessment of disease severity, these methods require that images are taken in the shade, either under an umbrella or dense plant canopy, to minimize the reflected surface light; in addition, it is preferred that these images be taken with a black background. A computer vision–based system, Cercospora leaf spot (CLS) Rater, was developed to rate the degree of CLS infection of sugar beet on a scale of 0–10 using plant‐level images taken in the field (Atoum et al., 2016). Barbedo (2014) designed an automatic image analysis system to detect and quantify disease symptoms in a variety of leaves with different shapes and sizes. This innovative program was able to successfully quantify the disease severity from images of multiple leaves in less time than standard methods. However, all of these published methods have limited ability to distinguish a specific disease based on a large number of overlapping biotic and abiotic stress symptoms.

The most common foliar diseases of apples are apple scab, powdery mildew, fire blight, Alternaria leaf spot, and frogeye leaf spot. Apple scab, caused by the fungal pathogen *Venturia inaequalis*, is one of the most economically important fungal diseases of apples in temperate regions of the world (MacHardy et al., 2001). The typical symptoms of apple scab are visible fungal structures on the leaf and fruit surface (Fig. 1A). The initial infection appears as black or olive‐brown lesions bulging on the leaf’s adaxial surface; later stages show chlorotic sporulating lesions on infected leaves. Apple scab affects not only leaves, but fruits as well, and can cause premature fruit and leaf fall and fruit deformation, with infected fruits showing dark‐colored, sharply bordered, brown, and corky lesions. Cedar apple rust is caused by the Basidiomycotina fungus *Gymnosporangium yamadai miyabe*. Early symptoms of the disease are small, light yellow spots on leaves that later expand and turn bright orange. The infected leaves become swollen, enlarged, and curled at the edges; in severe cases, premature dropping of leaves occurs. Severe outbreaks of rust pathogen lasting two to three years can severely injure or kill the trees of susceptible apple varieties (Jones and Bartholomew, 1915). Other foliar diseases of apples, especially Alternaria leaf spot and frogeye leaf spot, can cause similar‐appearing symptoms that can be difficult to distinguish without expert and careful inspection, and various studies have presented methods to distinguish among them. A CNN model was used to identify four apple leaf diseases (mosaic, rust, brown spot, and Alternaria leaf spot) with an overall accuracy of 97.62% (Liu et al., 2018). Dubey and Jalal (2016) used a *k*‐means clustering method for the detection of infected tissues and support vector machines to classify healthy and infected apple fruits based on color, texture, and shape. The multi‐class support vector machine was reported to have the potential to successfully categorize apple fruit into healthy or infected categories using features extracted from fruits (Dubey and Jalal, 2016).

In this study, we have created an expert‐annotated pilot data set for apple scab–infected, cedar apple rust–infected, and healthy leaves. We used this data set to train and test a CNN model for disease classification and as the basis of a Kaggle competition—the Plant Pathology Challenge (https://www.kaggle.com/c/plant‐pathology‐2020‐fgvc7)—as a part of the Seventh Fine‐Grained Visual Categorization (FGVC7) workshop at the 2020 Computer Vision and Pattern Recognition conference (CVPR 2020).

## METHODS

### Apple foliar disease data set and annotation

We captured high‐quality, real‐life RGB images of multiple apple foliar disease symptoms during the 2019 growing season from commercially grown cultivars in an unsprayed apple orchard at Cornell AgriTech (Geneva, New York, USA). Photos were taken using a Canon Rebel T5i DSLR (Canon Inc., Tokyo, Japan) and smartphones under various illumination, angle, surface, and noise conditions (Figs. 2, 3). The complexities of the data set were increased by including (1) an imbalanced data set of different disease categories, (2) non‐homogeneous image backgrounds, (3) images taken at different times of day, (4) images from plants at different maturity stages, (5) images displaying multiple diseases, and (6) images taken using different focus settings. A majority of the pictures taken were of apple scab, cedar apple rust, Alternaria leaf spot, frogeye leaf spot, and healthy leaves.

We created an annotated disease data set by manually annotating images of cedar apple rust (Fig. 3A), apple scab (Fig. 3B), and healthy leaves (Fig. 3D), using the unique and easily distinguishable symptomatic features of these diseases. An expert plant pathologist confirmed the annotations, particularly for images that were difficult to differentiate symptomatically (e.g., Alternaria leaf spot and frogeye leaf spot) and for complex disease symptoms caused by multiple diseases (Fig. 3C) of similar appearance on the same leaf. The data set was randomly split into training (80%) and stratified test sets (20%), making sure that all four disease categories were represented in both data sets.

### Disease classification using a standard CNN

We trained an off‐the‐shelf CNN on this pilot data set for classification of apple scab, cedar apple rust, complex disease symptoms (leaves with more than one disease in the same leaf), and healthy leaves. Specifically, we took a ResNet50 network pre‐trained on ImageNet (Russakovsky et al., 2015; He et al., 2016) and fine‐tuned the network weights on our annotated disease data set.

### Plant Pathology Challenge for the CVPR 2020 FGVC7 workshop

This expert‐annotated pilot data set for apple scab, cedar apple rust, complex disease symptoms, and healthy leaves was made available to the Kaggle community for the Plant Pathology Challenge competition as a part of the FGVC7 workshop at CVPR 2020. In addition to the images, a file with each image_id from the test set was also submitted to predict a probability for each target variable. The competition was launched on Kaggle and made available to the global community to compete on 9 March 2020 and was open for final submission until 26 May 2020. The accuracy of submitted models to classify diseases was evaluated based on mean area under the ROC curve (AUC) values.

Participants in the 2020 Kaggle competition were asked to train a model using images from the training data set to (1) accurately classify a given image from the testing data set into different disease categories or as a healthy leaf; (2) accurately distinguish between various diseases, including for cases where more than one disease appeared on a single leaf; (3) develop an algorithm for quantification of disease severity from the images taken under real‐life variable conditions; (4) deal with rare classes and novel symptoms; (5) address depth perception (i.e., angle, light, shade, physiological age of the leaf); and (6) incorporate expert knowledge in identification, annotation, quantification, and guiding computer vision to search for relevant features during learning. These same objectives will apply to future Kaggle competitions.

## RESULTS

### Apple foliar disease data set and annotation

The pilot data set consists of 3651 high‐quality annotated RGB images showing symptoms of cedar apple rust (Fig. 3A) and apple scab (Fig. 3B), as well as leaves displaying complex disease symptoms (i.e., more than one disease on the same leaf; Fig. 3C) and healthy apple leaves (Fig. 3D). Images represent real‐life field scenarios and were taken under various illumination, angle, surface, and noise conditions (Figs. 2, 3), as described above. We also have a large number of images of Alternaria leaf spot and frogeye leaf spot that still need to be annotated. In addition, there are a number of images that were taken later in the season, as well as images of leaves with multiple similar‐looking symptoms creating a complex of symptoms, that still need expert confirmation and annotation (Fig. 3C). Of the 3651 RGB images, there are 1200 of apple scab, 1399 of cedar apple rust, 187 of complex disease symptoms (i.e., more than one disease on the same leaf), and 865 of healthy leaves. Among 3651 RGB images in the initial data set, seven duplicate images were found and were deleted from the final image data set. The final data set was split into a training data set with 2921 images (80%) and a test data set with 723 images (20%).

### Disease classification using a standard CNN

The overall test accuracy achieved by a ResNet50 network pre‐trained on ImageNet was 97% (i.e., 97% of test images were correctly categorized), with the network achieving high accuracy predictions on most categories. The exception is the “complex disease symptoms” category, which was classified with just 51% accuracy. The algorithm is available on GitHub (https://github.com/rthapa‐26/FGVC‐Plant‐Pathology‐2020‐challenge‐dataset‐).

### Plant Pathology Challenge for the CVPR 2020 FGVC7 workshop

**Figure 4:**
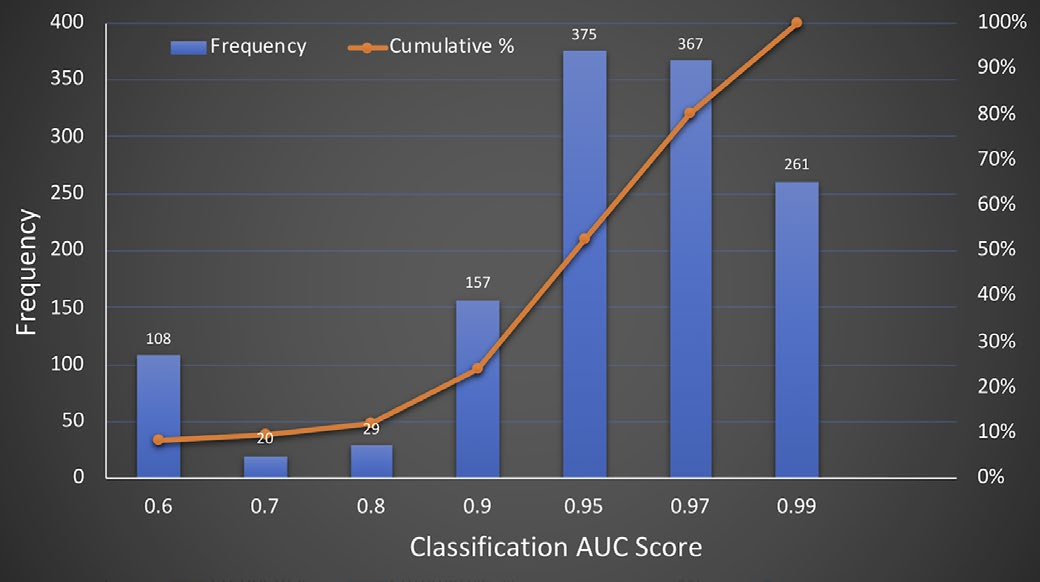
Frequency distribution of entries showing area under the ROC curve (AUC) values from each team. The number above the blue bar shows the total number of teams reporting the AUC score and the red line shows the cumulative percentage of the total teams reporting the corresponding AUC scores.

The expert‐annotated pilot data set of 3644 images was made available to the Kaggle community for the Plant Pathology Challenge competition as described above. A total of 1317 teams participated and submitted around 22,551 model entries in the competition. In order to avoid overfitting of the models, Kaggle pre‐divided the test data set into a public data set and a private data set. The public data set was provided to the participants while the private data set was kept hidden until the end of the competition. The results of the public leaderboard showed that ~28% of teams had entries between 0.95 and 0.97 AUC (Fig. 4), and 20% of teams had top entries above 0.97 AUC. More than 250 contending teams reported an AUC value greater than 0.985. The AUC values on the private leaderboard were used to identify the three highest‐scoring teams, with AUC values of 0.98445, 0.98182, and 0.98089, respectively.

## DISCUSSION

We have developed an expert‐annotated data set with 3651 high‐quality images of symptoms of apple scab, cedar apple rust, complex disease symptoms, and healthy leaves. A curated data set of disease symptoms, paired with machine learning algorithms, offers a great opportunity to develop an active scouting system for more accurate and timely disease management (Sankaran et al., 2010). For example, initiatives like PlantVillage have emerged to collect, host, and share large and diverse data sets for use in computer vision to identify diseases (Mohanty et al., 2016). A critical limitation is that the majority of the >50,0000 images collected by PlantVillage have homogeneous backgrounds (Mohanty et al., 2016) that do not accurately reflect conditions and symptoms observed in real‐world scenarios. In fact, researchers observed a decrease in accuracy when models trained with images from PlantVillage were applied to predict the accuracy of images collected from other online resources (Mohanty et al., 2016; Nestsiarenia, 2018). This suggests that images of single leaves from controlled experiments using uniform light conditions, few diseases, and distinct disease symptoms do not reflect real‐life scenarios in growers’ fields.

Early detection of disease symptoms can be used to schedule pesticide application and pruning of infected tissues or eradication of infected apple trees to reduce the chance of severe outbreaks (Norelli et al., 2000; Delalieux et al., 2007). Current disease identification practices in apple orchards involve scouting by experienced pathologists, shipping of potentially infected samples with visible symptoms to remote laboratories for chemical and PCR tests (Brown et al., 1993; Li et al., 1994; Johnson et al., 2000), and sharing digital images with experts for identification and recommendation. During a busy growing season, the time required for observation, sample collection, shipping, analysis, and identification often results in growers not receiving recommendations until it is too late to take the necessary control measures. There are also a limited number of extension experts who can provide in‐person and on‐site identification. Disease diagnosis can be a particular challenge for inexperienced growers who lack expertise with the crop or for home growers, whose trees can be a source of pathogen spread to commercial orchards (Beckerman et al., 2013).

Deep learning methods such as deep CNNs are yielding unprecedented accuracy on visual recognition tasks (Krizhevsky et al., 2012; Donahue et al., 2014; Yan et al., 2015; Hatami et al., 2018). However, the problem of disease recognition is distinct from standard deep learning applications where there are multiple observations of each object from varying angles. The visual features that define a disease can be small or subtle, localized, and symptoms of more than one disease are often present on a single leaf (Barbedo, 2016). Our pilot data set consisting of thousands of high‐quality, expert‐annotated images, including both healthy leaves and leaves exhibiting symptoms of apple scab and cedar apple rust, provides an opportunity to test standard deep learning algorithms. A CNN trained on this data set can achieve 97% test accuracy, giving us confidence in the use of this approach for disease classification. The lower prediction accuracy (51%) of the deep learning method for images with multiple diseases on a single leaf highlights the need to improve methods for image capture and segmentation to increase disease classification accuracy. We need to (1) handle a broader class of diseases than the three classes in our pilot data set, (2) generalize to more diverse image conditions than are present in our pilot data set, (3) achieve even greater than 97% accuracy for a practical system, and (4) visually assess properties beyond presence of disease, such as degree of severity. The performance metric depicted by the overall accuracy value of our results is relatively lower than the accuracy value reported by Liu et al. (2018). Although Liu et al. (2018) used four foliar diseases of apple (i.e., Alternaria leaf spot, apple mosaic virus, cedar apple rust, and brown spot), their higher accuracy value (97.62%) could be caused by the distinct symptoms of these diseases and by the images being taken under controlled conditions that do not reflect the complexities of a natural scenario. The images in Liu et al. (2018) used varying illumination levels and were rotated at different angles to generate multiple pathological images, mimicking different light distributions in a natural setting. In an actual orchard, additional variables can increase complexity and noise in the data set. The most common variables are: (1) non‐uniform image backgrounds, (2) the presence of additional disorders caused by physiological factors, cold and heat stress, or mechanical and insect damage that are difficult to distinguish from disease symptoms (Barbedo, 2016), (3) imaging device used, and (4) image capture conditions (e.g., distance from the leaf, illumination, wind).

**Figure 5:**
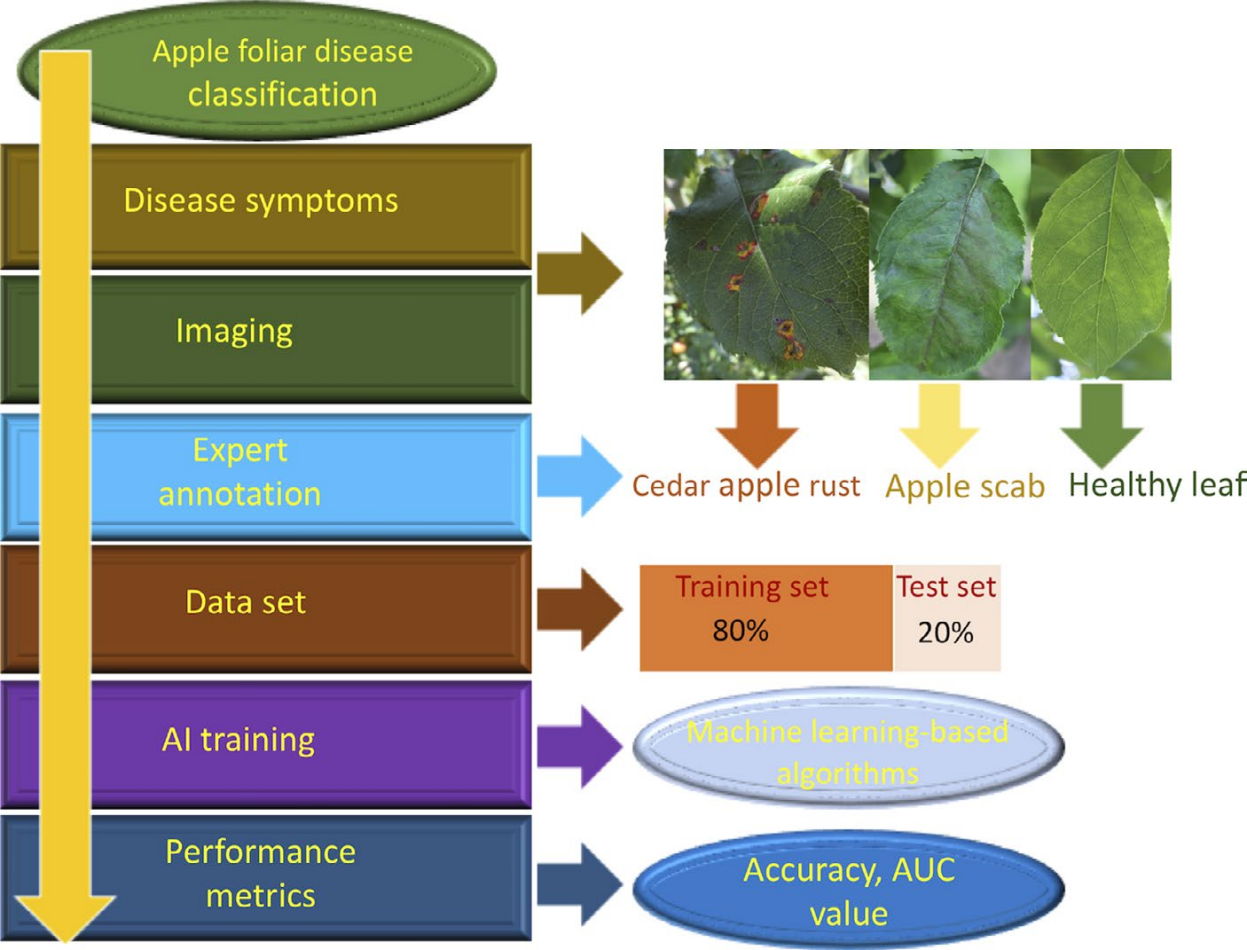
A flow diagram of the steps to develop computer vision models for disease detection based on annotated leaf images.

Our pilot data set was made available on the Kaggle platform for the Plant Pathology Challenge competition during the FGVC7 workshop at CVPR 2020 to train disease classification models (Fig. 5). In about two and a half months, 1317 teams participated, submitting approximately 22,551 entries. Among all the submitted entries in the competition, the highest AUC value in the private leaderboard was reported to be 0.98445. The large number of submissions with high accuracy demonstrate the promise of this method for automated plant disease diagnosis. A previous plant disease–oriented challenge organized through the FGVC6 workshop at CVPR 2019 was “iCassava” (Mwebaze et al., 2019). The goal of iCassava was to build a robust computer vision algorithm to distinguish between similar‐appearing foliar diseases in cassava leaves and to categorize the severity levels of the diseases. A data set with images of the four diseases—cassava brown streak disease, cassava mosaic disease, cassava bacterial blight, and cassava green mite—was provided to a Kaggle competion. The complexity of the data set was increased by including (1) images with different backgrounds and scales, (2) images taken at different times of day, (3) some images with poor focus, and (4) images representing mutiple co‐occuring diseases on the same plants, as well as by including more unlabeled images (12,595) than labeled images (9436) (Mwebaze et al., 2019). These complexities of the data set might have contributed to their relatively lower accuracy of 93%. The models trained on this data set are likely to have further reduced accuracy when applied to images collected in a farmer’s field with a large number of real‐world variables. The relatively small size of the data set might have resulted in the high accuracy scores of the models. However, the images of the foliar symptoms in the data set were captured from a large number of apple cultivars over four months under different illumination conditions and represented visually diverse symptoms, from early to late stage of infection on young and old leaves in each disease category. Additionally, we have randomly assigned 80% and 20% of images to the training and test data sets to decrease the bias toward factors such as similar cultivars, leaf types, and imaging conditions.

Going forward, our data set will continue to contribute toward advancing the state of the art in automatic image classification for identification and quantification of diseases from a large number of symptom classes in various real‐world scenarios. The high participation in the competition showed that there are many image processing and machine learning experts who can offer innovative solutions to plant disease classification. The major limitation is not the lack of interest but rather unavailability of annotated disease data sets. There are very few existing databases and, in most cases, they are limited to the biological science community. The open source database PlantVillage (https://www.plantvillage.org/) has more than 50,000 images of healthy and infected leaves, but these images are taken using a uniform background, which does not accurately reflect the the complex disease symptoms found in growers’ fields. The foliar disease symptom images in our image data set represent the complexities that potentially exist in real‐life scenarios. The machine learning models developed using such databases will potentially reduce overfitting of models and could be efficient for accurate classification of apple diseases. We will continue adding more images captured using a range of angles, lighting, and distances to our pilot data set to build an even larger, more comprehensive expert‐annotated data set. This will include manually capturing and annotating images of symptoms on apple leaves representing apple scab, fire blight, powdery mildew, cedar apple rust, Alternaria leaf spot, frogeye leaf spot, and Marssonina leaf blotch, as well as insect damage (e.g., apple aphids and mites) on leaves. We will also capture and annotate images of fruit with apple scab, bitter rot, and brown rot.

## Data Availability

The disease classification algorithm is available on GitHub (https://github.com/rthapa‐26/FGVC‐Plant‐Pathology‐2020‐challenge‐dataset‐). The image dataset is freely available to download on Kaggle (https://www.kaggle.com/c/plant‐pathology‐2020‐fgvc7/data).
